# A Novel IoT-Enabled Wireless Sensor Grid for Spatial and Temporal Evaluation of Tracer Gas Dispersion

**DOI:** 10.3390/s23083920

**Published:** 2023-04-12

**Authors:** Tsz-Wun Tsang, Kwok-Wai Mui, Ling-Tim Wong, Kwok-Yung Law, Ka-Wing Shek

**Affiliations:** 1 Department of Building Environment and Energy Engineering, The Hong Kong Polytechnic University, Hong Kong, China; 2 Department of Mechanical Engineering, The Hong Kong Polytechnic University, Hong Kong, China

**Keywords:** tracer gas system, dispersion, airflow, Internet of Things (IoT), wireless sensing network (WSN)

## Abstract

Current IoT applications in indoor air focus mainly on general monitoring. This study proposed a novel IoT application to evaluate airflow patterns and ventilation performance using tracer gas. The tracer gas is a surrogate for small-size particles and bioaerosols and is used in dispersion and ventilation studies. Prevalent commercial tracer-gas-measuring instruments, although highly accurate, are relatively expensive, have a long sampling cycle, and are limited in the number of sampling points. To enhance the spatial and temporal understanding of tracer gas dispersion under the influence of ventilation, a novel application of an IoT-enabled, wireless R134a sensing network using commercially available small sensors was proposed. The system has a detection range of 5–100 ppm and a sampling cycle of 10 s. Using Wi-Fi communication, the measurement data are transmitted to and stored in a cloud database for remote, real-time analysis. The novel system provides a quick response, detailed spatial and temporal profiles of the tracer gas level, and a comparable air change rate analysis. With multiple units deployed as a wireless sensing network, the system can be applied as an affordable alternative to traditional tracer gas systems to identify the dispersion pathway of the tracer gas and the general airflow direction.

## 1. Introduction

The Coronavirus disease 2019 (COVID-19) pandemic has posed a significant threat to the global economy in recent years. Long-range airborne transmissions have been recognized as the potential model of disease transmission, asserting the importance of research in related fields [1,2,3,4,5,6,7]. Although the mechanism of pathogen-laden aerosol generation is well-documented [8,9,10], experimental data on dispersion in indoor environments are insufficient. The literature suggests a strong and significant association between ventilation, air movement, and the airborne transmission of infectious diseases [11,12]. Therefore, studying the effects of building ventilation on airborne transmission could provide helpful information in identifying potential transmission pathways and infection risks within the premises.

Building ventilation performance and its effects on airborne transmission can be evaluated computationally or experimentally. Despite the ability to simulate any physical conditions theoretically, computational fluid dynamics (CFD) models require validation using experimental or measurement results to ensure realistic and reliable simulations [13]. Some studies employed the tracer gas technique to simulate the diffusion of gaseous pollutants, fine particles, and bioaerosols. For example, Yu et al. [14] investigated the possible internal spread route between adjacent horizontal flats induced by air infiltration in a residential building. They used tracer gas as a surrogate for stack aerosols containing virus-laden droplets. Wang et al. [15] identified a dispersion of tracer gas along two connected drainage stacks in a high-rise residential building which agreed with the distribution of infected households.

Notably, there is some argument about the suitability of gaseous tracers for representing fine, infectious aerosols. Indeed, tracer gases and aerosols differ in their physical characteristics and surface behaviour [15]. Several existing studies attempted to investigate the suitability and effectiveness of a tracer gas for simulating the movement of small particles. For instance, Bivolarova et al. [16] experimentally compared the dispersion and the distribution of tracer gas and 0.7 μm aerosols under various ventilation schemes in a single-bed hospital room. Influenced by the free convection flow and ventilation airflow but not the effect of buoyancy, the tracer gas behaved the same as the small-size particles, making it a preferable analogue to bioaerosols. Gao et al. [17] developed numerical models to investigate the airborne transmission between flats in high-rise residential buildings and found that 1 μm fine particles disperse similarly to gaseous pollutants. In their position paper, Ai et al. [18] supported the notion that a tracer gas is a suitable surrogate for studying airborne transmission in the built environment as the aerodynamics of the fine exhalation droplet nuclei are close to that of a gas molecule. Using tracer gas to experimentally or computationally simulate exhaled droplet nuclei is preferable due to its lower complexity and lower demand for user knowledge.

Despite the importance of having experimental data on tracer gas dispersion for understanding bioaerosol transportation in indoor environments, existing studies could only produce temporal and spatial understandings with low resolutions due to high sampling costs. The literature review on tracer gas experiments indicated that the multipoint sampler and doser (INNOVA 1303) and the photoacoustic gas monitor (INNOVA 1412) produced by LumaSense Technologies appear to be the most popular and the only tracer gas systems currently available in the market for such a purpose. Given their high accuracy, based on the infrared photoacoustic spectroscopy technique, these commercial instruments have the drawbacks of a long data acquisition interval of around 40 s, limited sampling channels (six channels), and a maximum sampling point distance of 50 m from the instrument [19]. If the multipoint sampling of all six channels is adopted, the entire sampling cycle can be as long as 4.5 min, i.e., the sampling interval for each point = 4.5 min [20].

The need for affordable instruments for tracer gas measurement has prompted the demand for fast-response, mobile, and compact devices without constraints on the number of sampling points and locations. This is especially important for studies investigating airborne transmission pathways in indoor environments, as high-frequency multipoint sampling is essential. In this regard, this study developed a novel Internet of Things (IoT) -enabled wireless sensing network (WSN) to identify tracer gas dispersion and analyse ventilation performance rapidly. Unlike other indoor air IoT applications that focus on the general monitoring of air contaminants, this proposed IoT application was developed for experimental purposes to identify tracer gas dispersion and evaluate ventilation performance. This IoT-enabled WSN was built upon existing IoT development with a new sensor application. Adjustments to the IoT system and the development of a cloud server were carried out for system compatibility, real-time device control, data visualization, analysis, storage, and retravel. Its performance was assessed by comparing it with commercial measuring instruments: the Brüel & Kjær (B&K) 1302 Multi-gas Monitor and the INNOVA 1303 Multi-point Sampler and Doser with an SF_6_ filter. The systems obtained and compared the spatial–temporal distributions of the SF_6_ and R134a under different ventilation schemes. The effectiveness of the novel system in assessing ventilation performance was also evaluated. This study aims to provide an economical and quick solution to identifying airborne transmission pathways in indoor environments, which can be very useful for facility management to formulate mitigation strategies to combat airborne diseases during pandemics.

## 2. Materials and Methods

### 2.1. IoT Applications in Indoor Air

With the advancement in IoT technologies and low-cost sensors, increasing IoT applications can be adopted in indoor environments for environmental monitoring and evaluation. Saini et al. [21] systematically reviewed the current low-cost sensing technologies for IoT IAQ monitoring. Chojer et al. [22] also examined the recent development of low-cost IAQ monitoring devices and summarized the monitoring parameters presented in the studies. Carbon dioxide (CO_2_) was the most common IAQ parameter included in these systems, with others also measuring the levels of particles and some gaseous pollutants. It is noteworthy that from the literature, the IoT applications for indoor air mainly focus on general monitoring. Some IAQ monitoring devices developed for the early warning of airborne infection were based on the notion that specific IAQ parameters can be surrogates of the airborne infection risk [23]. Despite different objectives, the applications were built upon assessing the levels of IAQ pollutants in indoor environments.

The novel method for tracer gas experiments using a wireless communication method presented in this study could solve the limitations of existing tracer gas measurement instruments. Specifically, this study proposed an application of IoT-enabled wireless sensing networks for evaluating airflow patterns and ventilation performance. Instead of measuring the indoor air pollutants, tracer gas would be released, and the proposed wireless sensing network would monitor its dispersion. Notably, the study’s novelty lies in applying an R134a sensor using a developed IoT system rather than developing the IoT system itself. To ensure system compatibility for the specific sensor, fine-tuning the IoT system and developing a cloud server for system control, data retrieval, storage, analysis and visualization were carried out. The study aimed to develop an economical, quick, and high-resolution tracer gas monitoring system to identify airborne transmission pathways in indoor environments. The system adopted commercially available Wi-Fi modules and sensors and can assess the spatial and temporal variations in tracer gas levels facilitated by the application of the IoT. More specifically, the IoT technologies adopted in this system include wireless data transmission through the internet, the cloud computing of sensor signals, and real-time device control through a web-based control platform. 

### 2.2. Background of Different Tracer Gases

An ideal tracer gas must be safe, non-reactive, insensible, unique, and measurable [24]. Among all commonly used tracer gases, sulphur hexafluoride (SF_6_) gas is frequently used in tracer gas experiments due to its low toxicity and the fact that it is non-existent in typical indoor environments. A fast-response and economical SF_6_ sensor was recently seen in the literature but is not yet commercially available [25]. Nitrous oxide (N_2_O) is sometimes used as a tracer as it is physically similar to CO_2_, a respiratory contaminant that can be used as a proxy for infection risks [26]. However, N_2_O, commonly known as laughing gas and used for anesthetic and pain-reducing purposes, could cause short-term mental impairment and potentially neurological damage [27]. In a few studies, 1,1,1,2-Tetrafluoroethane (R134a) was adopted to evaluate the effectiveness of various ventilation strategies [20,28,29,30]. R134a was also used in other experimental studies investigating air distribution and ventilation effectiveness [31,32].

R134a, one of the common tracer gases for studying airflows in buildings [33], is a hydrofluorocarbon and haloalkane-based refrigerant. It has insignificant ozone depletion potential and is expected to volatilize within days to weeks. It also does not affect human health, even at high concentrations, and does not accumulate in biota or adsorb to soil or sediment [34,35]. Although it is considered a greenhouse gas, as other common tracer gases are [36], this application only utilizes a small amount of R134a, which will have negligible environmental effects.

There is no limitation on which type of tracer gas sensor is to be incorporated into the module. Although the measurement technology of commercially available, small CO_2_ sensors is more well-developed than that of the R134a sensors, the development of a novel tracer gas system that aims to identify the subtle changes in the levels as a means of detecting the dispersion and distribution of the tracer gas as a surrogate for bioaerosols cannot be achieved using CO_2_ as a tracer as there are always background levels of CO_2_ that can be affected by the presence of occupants [37]. In addition, CO_2_ sensors generally have a ±50 ppm accuracy, which is relatively large for such purposes. In contrast, R134a is non-toxic and rarely exists in the background. The current study adopted an R134a small sensor to develop the wireless tracer gas system. 

### 2.3. Development of the R134a Tracer-Gas-Sensing Network

#### 2.3.1. Tracer-Gas-Sensing Network with Calibrated R134a Sensor

Despite their accuracy, one of the significant drawbacks of traditional R134a-measuring instruments is the high price. Thus, there is a limited number of sampling points. The commercial R134a sensor FIGARO TGS 3830 was adopted in this novel system due to its low cost and availability. This sensor uses a tin dioxide (SnO_2_) semiconductor as the sensing element, which is susceptible to R134a (≤0.85 change ratio of the sensor’s resistance (Rs)), and the relative change in conductivity reflects the change in the gas concentration. As the sensor is designed to detect refrigerant leaks, it has a quick response time and a range of 5–100 ppm [38]. For spatial tracer gas detection to identify the airborne transmission pathways and to evaluate the ventilation performance, relative values at different locations and the changes in concentration over time are adequate. The calibration was conducted by placing all tracer gas receivers in the sensing network nearby in a mechanically ventilated room with six air change rates (ACH) and complete air mixing. R134a gas was dosed for 15 s so that the peak concentration inside the room reached 100 ppm. The R134a concentration will decrease gradually over time from ventilation, and the receivers should supposedly detect the same spatial and temporal variations of R134a. One of the receivers was used as the reference standard, and the best-fitting function thus determined the correlations between the data collected by other receivers and the reference. 

The sensing device was designed to be powered by an AC/DC adapter (output voltage: 5 V; output current: 2 A) or a rechargeable 36,300 mAh lithium-ion battery that can support continuous measurement for up to 3 days. A traditional tracer gas sampler is equipped with an internal pump such that active sampling can be carried out in spaces with large volumes to draw the air from the sampling point into the gas analyser. On the contrary, for the system developed using IoT technology, each receiver has a sensor for detection. Therefore, the wireless sensing network does not require an internal pump for active sampling. Instead, it relies on air diffusion, driven by indoor airflow, to provide more detailed variations in the tracer gas concentration, which is influenced by ventilation. A poly(methylmethacrylate) (PMMA) case with an air vent and an aluminium cover was used as housing to ensure adequate air diffusion, heat dissipation, and robustness. Notably, this study intended to present and evaluate a novel IoT application for assessing airflow patterns and ventilation performance in indoor air. The development process, technologies, and the system architecture were only briefly described for brevity.

#### 2.3.2. Sampling, Data Transfer, Storage, and Real-Time Data Analysis

**Figure 1:**
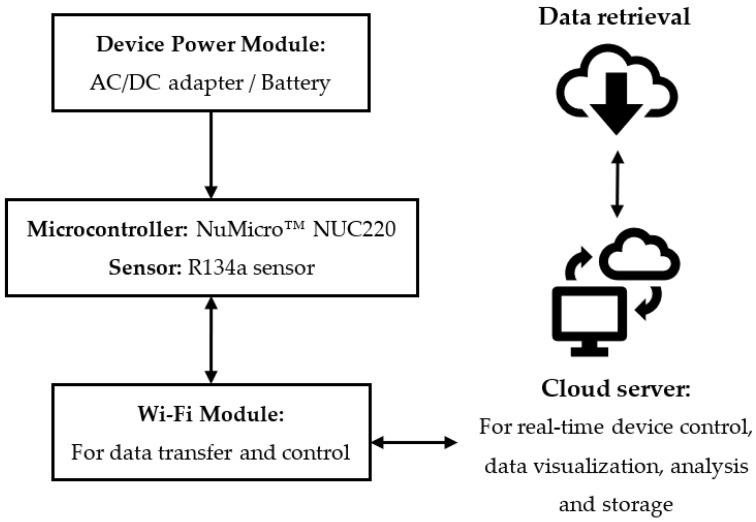
Operational diagram of the tracer gas system.

The operational diagram of the tracer gas system is shown in Figure 1. The receiver was programmed to collect R134a data every 10 s. The data was immediately transmitted wirelessly to a cloud database through a 2.4 GHz ESP8266 Wi-Fi module, a low-cost wireless transceiver developed by Espressif Systems for endpoint IoT applications [39]. Data pre-processing, including signal conversion and data adjustment, was performed in the cloud using a cloud-structured query language (SQL).

Traditional tracer gas systems require on-site setup and operation, which may be problematic if the premises are unsafe for human occupancy. This novel system was designed to be operated remotely. With the help of remote-control robots, the system can be delivered to a hazardous environment and placed at designated locations. The entire operation and control of the system, including monitoring and configuring the devices, setting up and modifying the tracer gas experiment, and visualizing and analysing the measurement data in real-time, can be conducted off-site using a web-based tracer gas system control platform also developed in this study.

### 2.4. Experiment Setup for Systems Comparison

To evaluate the performance of the novel tracer gas system in identifying the tracer gas dispersion pathways in an indoor environment under the influence of ventilation, laboratory tests were carried out with different ventilation configurations. The system’s performance was compared to the typical Brüel & Kjær (B&K) 1302 Multi-gas Monitor and the INNOVA 1303 Multi-point Sampler and Doser, hereafter referred to as the reference system, in terms of the response time, sensitivity, accuracy, and resolution in evaluating the spatial–temporal variations in the tracer gas and accuracy in determining the air change rate. Three sets of experiments were carried out.

#### 2.4.1. Spatial and Temporal Variation

**Figure 2:**
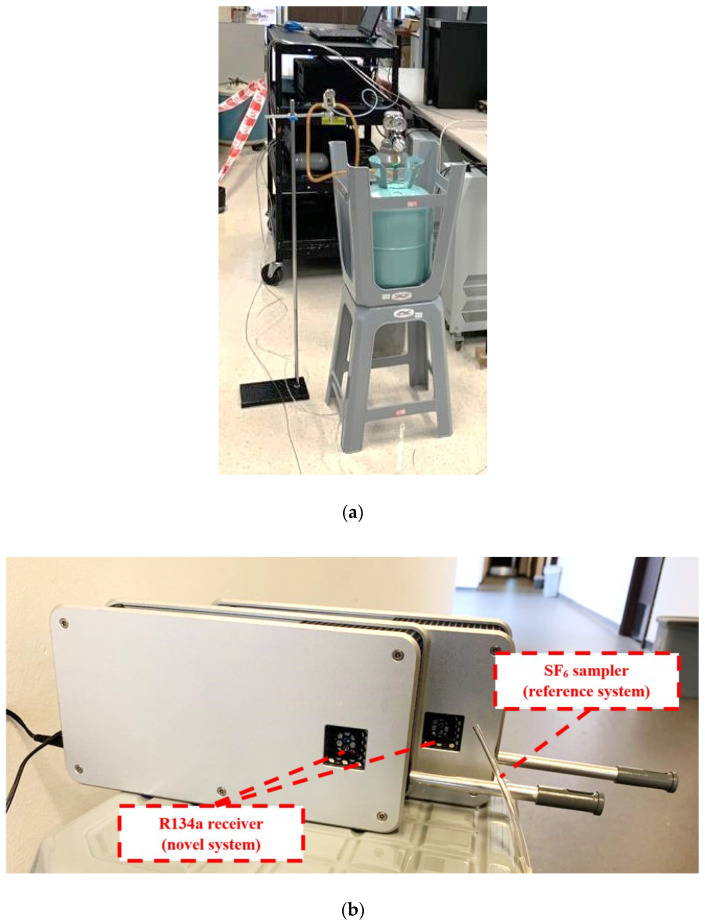

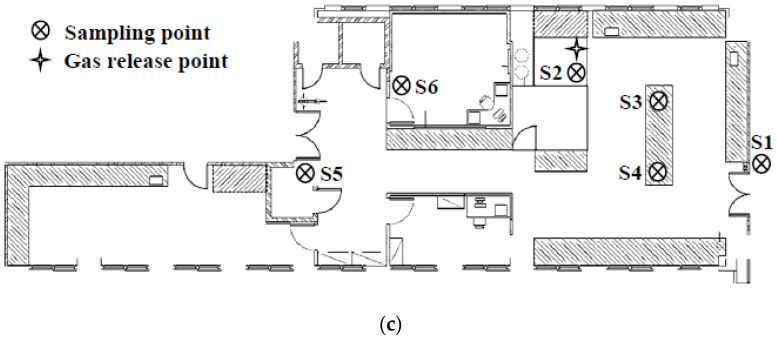
(**a**) Photograph of the experimental setup for the release of SF_6_ and R134a; (**b**) photograph of the sampling tube for SF_6_ measurement (the reference system) and the receivers for R134a measurement (the novel system); (**c**) layout of the testing laboratory with sampling and gas release point locations.

The first experiment aimed to identify the two systems’ sensitivity and detailedness in detecting spatial and temporal variations in the tracer gas concentration. SF_6_ and R134a were released at the same time at the exact location. The reference and novel tracer gas systems were then monitored at 6 locations in the laboratory. Although SF_6_ and R134a have slightly different densities (6.17 kg/m³ and 4.25 kg/m³) and molar masses (146.06 g/mol and 102.03 g/mol), both are small gas molecules that behave very similarly to aerosol particles with a small size (<3 µm) [16]. SF_6_ and R134a were simultaneously released nearby in the laboratory for 30 s, assuming both gases followed the ventilation-driven airflow. Thus, the spatial distributions of the two gases were very similar. The sampling locations were selected carefully, with four points at various distances from the source and without a natural barrier (open area). One point was in a room located inside the laboratory with a partition wall that was served by the same mechanical ventilation system, and one point was located outside the laboratory, in the corridor with the door closed. A wireless sensing grid was constructed by placing two novel tracer gas receivers very close to each SF_6_ sampling point to simultaneously measure the concentration of SF6 and R134a. Figure 2a,b shows the photographs of the tracer gas experiment and sampling setup. Figure 2c illustrates the gas release and sampling point locations.

The dynamic time warping method (DTW) for comparing temporal sequences was adopted to analyse the similarity between the tracer gas concentration data measured by the nearby novel system receivers [40]. Given the two time series datasets *X* = (*x*_1_, *x*_2_, …, *x_n_*) and *Y* = (*y*_1_, *y*_2_, …, *y_m_*), *n* and *m* ∈ ℕ, a warping path *P* = (*p*_1_, *p*_2_, …, *p_l_*), *l* ∈ ℕ indicates the alignment of the two sequences. The quality of the warping path can then be evaluated using the cost function *c_p_*, calculated using Equation (1) [41]. A lower cost function suggests a higher similarity between the two datasets.

(1) $$c_{p}\left(X,Y\right)=\sum_{l=1}^{L}c\left(x_{n_{l}},y_{m_{l}}\right)\;$$

The performance of the novel system in identifying the spatial distribution of tracer gas was evaluated by correlation, in which the correlation coefficient between the data collected by the novel and the reference systems at each sampling location can indicate the spatial–temporal coincidence of the data. A coefficient close to 1 indicates a high correlation.

#### 2.4.2. System Response Time and Ventilation Performance Evaluation

**Figure 3:**
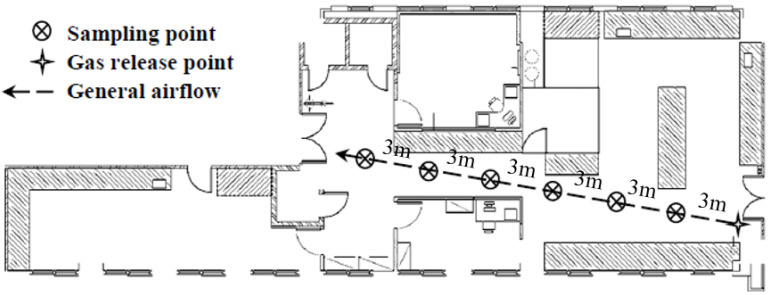
Layout of the testing laboratory with sampling and gas release point locations for system response time evaluation.

The second experiment tested the responses of the two systems in detecting a sudden increase in the tracer gas concentrations at various distances. The SF_6_ and R134a gas mixture was released upstream of the general airflow direction in the laboratory (i.e., a downwind direction). The systems were placed at 3, 6, 9, 12, and 15 m from the source along the airflow direction. The performance of the system response was determined by the time required for it to pick up the first increase in tracer gas concentration by sight. The test was repeated at least three times to ensure data consistency. Figure 3 shows the gas release point and sampling point locations. 

The final experiment compared the effectiveness of the two systems in responding to the gradual change in the tracer gas concentration caused by ventilation. Thus, continuous gas concentration monitoring was conducted under three ventilation rates with repeated experiments. The accuracy of the novel system in determining the ventilation performance was evaluated by the tracer gas concentration decay method suggested in Equation (2), where *C(t)* is the tracer gas concentration at time *t*, *C*_0_ is the tracer gas concentration at time *t* = 0, and *λ* is the air change per hour [42].

(2) $$C\left(t\right)=C_{0}\times e^{-\lambda t}$$

## 3. Results and Discussion

### 3.1. Identification of Spatial and Temporal Variation

**Figure 4:**
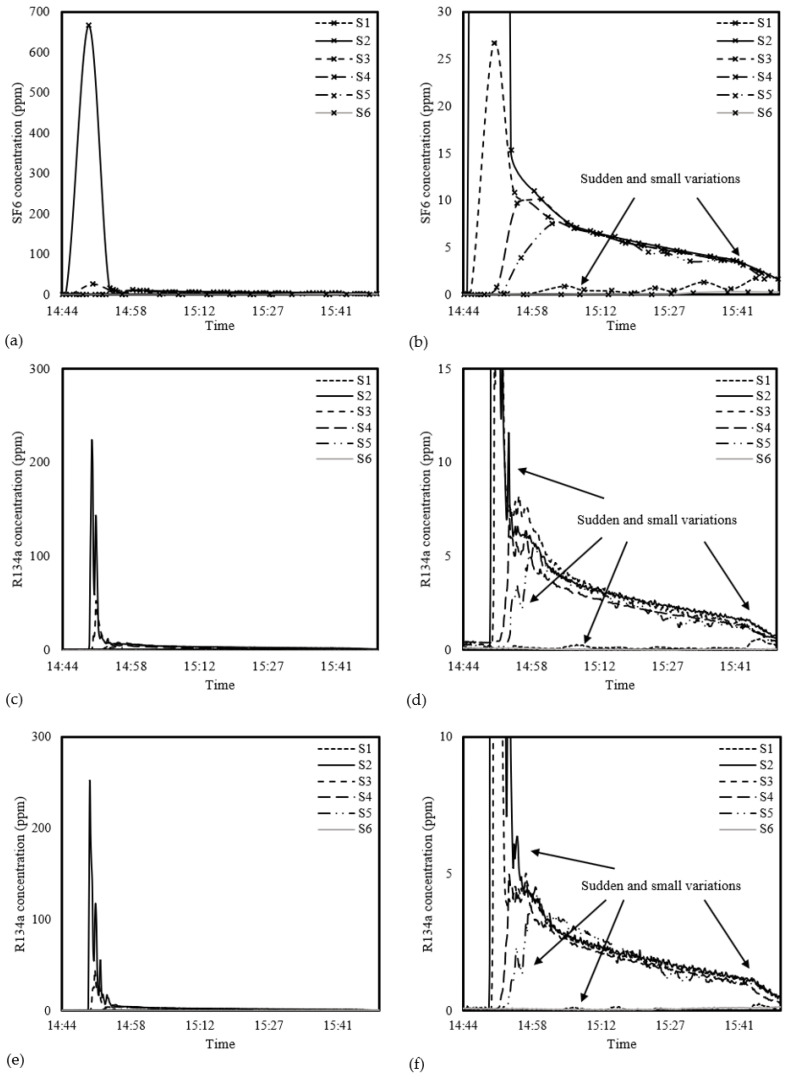
SF_6_ and R134a concentrations at six sampling points measured by the (**a**,**b**) reference system and (**c**–**f**) two novel system receivers at (**a**,**c**,**e**) a complete tracer gas concentration range and (**b**,**d**,**f**) a smaller range for detailed comparison.

Since the development of IoT-enabled WSNs for monitoring the dispersion of tracer gas is novel, more than a direct comparison of results from previous work is required. Some studies on developing low-cost IAQ monitoring devices validated the device’s performance through co-location tests using commercial counterparts [43,44,45]. Such a method of graphical comparison was also adopted in this study for performance evaluation. Figure 4 demonstrates the temporal–spatial variations in the tracer gas in the first experiment. Notably, since the sampling cycle of the multipoint sampling of the reference system was more than 4 min, there were only around 14 data points for each sampling point throughout the 1 hr experiment. On the other hand, the novel system collected over 350 data points at each point, with a sample cycle of 10 s. Thus, smooth lines connect the sampling data for a better visual comparison. 

Since the concentrations of SF_6_ and R134a released were not the same, instead of comparing the absolute concentration of the gases at various sampling points, the relative changes in the spatial and temporal concentration of the two gases could indicate the performance of the two systems. Figure 4a,c,e shows the variations in the tracer gases at a complete concentration range. For sampling point 2 (S2) of the reference system, the peak was very high, reaching almost 670 ppm; this is 24.6 times the peak concentration (about 27 ppm) measured at a nearby sampling point, S3. On the other hand, for the novel system, the S2 peaks picked up by the novel system receivers were 237.3 ppm on average, which was only 4.9 times the S3 peaks at an average of 48.35 ppm. The differences can be explained by the reference system using an internal pump for sampling. 

In contrast, without the pump, the novel system relies on diffusion driven by indoor airflow. When the tracer gases were released near S2 and were thus still concentrated at that location, the reference system also sampled at S2 (it takes turns to sample the six points). Therefore, an extremely high peak of SF_6_ was detected by the reference system with an active sampler.

For a more detailed comparison of the patterns of the tracer gases at different sampling locations, Figure 4b,d,f shows the tracer gas profiles at a smaller concentration range. At S1 and S6, due to obstruction by partition walls and doors, only a slight increase in SF_6_ and R134a was observed. On the other hand, the sampling points located in open areas (i.e., S2–S5) detected much higher concentrations of the tracer gases. In the initial stage of the experiment, after the gas was released, similar variations in the tracer gases SF_6_ and R134a were detected by both systems, with relative concentrations in the order of S2 > S3 > S4 > S5. Eventually, they reached the same concentration approximately 20 min after the gases were released. 

At the beginning of the experiment, the SF_6_ level at S2 and S3 detected by the reference system appeared to increase before R134a did, which was a graphical misrepresentation of interpolation. The reference system could only detect the increase in the SF_6_ level 3 min after the gases were released due to the long sampling duration (i.e., the data points jumped from 0 ppm to 670 ppm, connected by an interpolation curve). On the other hand, the novel system determined the increase in R134a level precisely within a 10 s interval. 

From Figure 4d,f, it can be seen that many of the minor variations (indicated by arrows), as slight as a change of 0.04 ppm, were detected by the two novel receivers. Instead, the novel receivers produced rough curves with many slight variations, indicating their ability to pick up the subtle changes induced by the ventilation airflow. On the contrary, since the sampling cycle for the reference system was much longer, it could not pick up the changes driven by airflow between the samplings, indicated by a very smooth interpolation curve. The reference and novel systems detected similar spatial and temporal tracer gas distribution patterns. 

Table 1 exhibits the alignment costs of the warping paths of the time-series data collected by two sets of novel system receivers, denoted as Receiver B and Receiver C, located near the six sampling points. A lower cost function indicates a higher similarity. Looking into the individual system, the two receivers of the novel system displayed very similar patterns and levels of R134a at each location. For instance, the two receivers at S5 presented a high similarity with a low DTW alignment cost of 225.9. In contrast, high costs of 606.8 and 2051.8 were found between the receivers at S2 and S5, suggesting the tracer gas profiles produced by the two receivers were similar at S5, and the profiles at S2 and S5 were different. Notably, a certain degree of similarity was observed in nearby receivers, for example, at S3 and S4, indicating a similar temporal variation at these locations. A certain degree of similarity was observed for Receiver B at S5 and Receiver C at S3. This could be due to the flow pattern of this tracer gas, which depends mainly on the airflow induced by the ventilation. 

**Figure 5:**
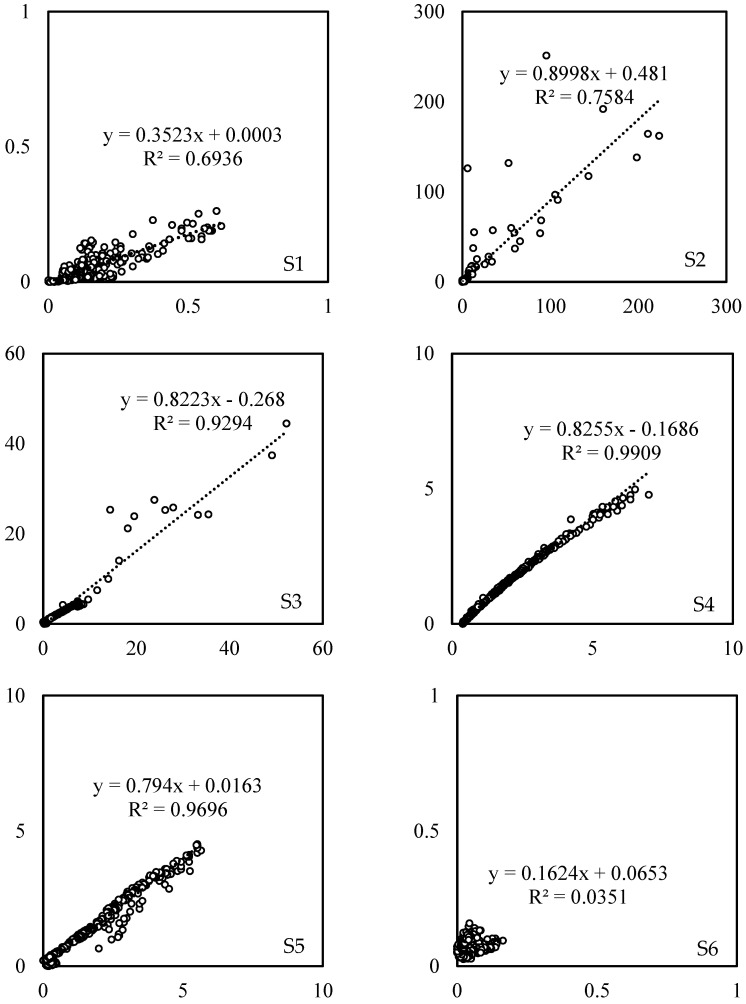
Correlations between R134a concentrations at six sampling points measured by the two novel system receivers. S1 to S6 are the sampling locations.

The correlations between the data measured by the nearby Receiver B and Receiver C at each sampling point were also examined to evaluate the performance of the receivers in the sensing network. From Figure 5, a high degree of linear correlation between the R134a levels measured by the nearby receivers was observed at S3–S5, with R^2^ = 0.93–0.99, suggesting a consistent and conforming performance of the sensing network. At S1, S2 and S6, since some of the levels detected were out of the sensor detection range of 5–100 ppm [38], poor correlations at these sampling locations with extremely high and low R134a levels were expected.

Table 2 displays the correlation matrix between the three sets of data collected by the reference system and the two receivers. Since the reference system and the novel system collect different numbers of samples, the correlation could only be performed to the timestamp at which both systems collected data. High correlations were found between data collected at the same sampling point by the two systems except for S6. The observation at S6 can be explained by very low levels of tracer gases being detected by both systems (SF_6_ range: 0–0.3 ppm, mean: 0.1047 ppm and R134a range: 0–0.158 ppm and 0–0.164 ppm, mean: 0.0721 ppm and 0.0491 ppm), which could only be the sensor noises rather than actual responses towards the changes in the tracer gas concentration. Moreover, high correlations were also discovered between data collected at S2 and S3 and S4 and S5, suggesting that the tracer gas concentration in the vicinity eventually reached equilibrium by ventilation mixing.

### 3.2. System Response

**Figure 6:**
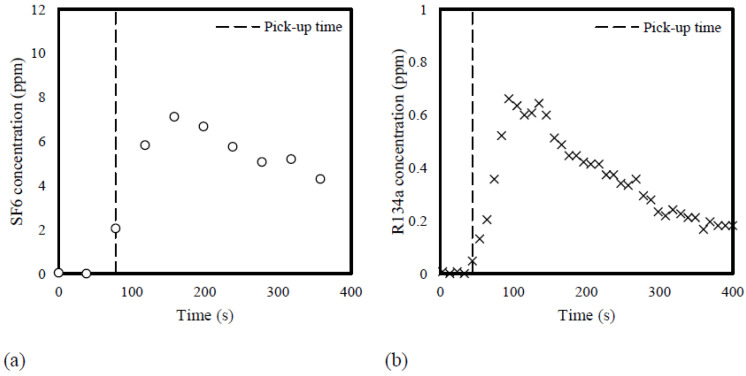
Tracer gas concentrations measured by (**a**) the reference system and (**b**) the novel system at 3 m away from the release point. Gas release time: time = 0 s.

The system responses were evaluated by the time required for the two systems to detect the first and slightest increase in tracer gas concentration at various distances from the gas release point. Since the novel system collects data every 10 s while the reference system has the shortest sampling cycle of approximately 40 s for a single-point measurement, the novel system demonstrated a 26.5% faster rate on average in detecting the increase in gas concentration at closer distances from the source (≤12 m). At a far distance, the responses of the two systems were comparable. Figure 6 illustrates the tracer gas profiles during one of the tests at 3 m. It can be seen that since the novel system has a much shorter response time and a high sensitivity as the system detected a slight increase of 0.05 ppm R134a at 44 s after the release, while the reference system took 78 s to sense the change in the SF_6_ level.

**Figure 7:**
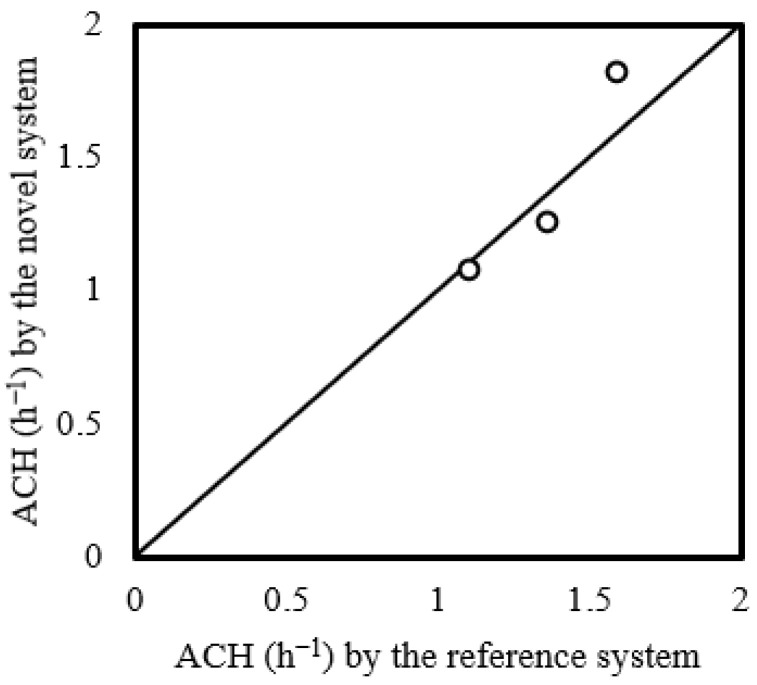
Air change rates (ACHs) evaluated using data collected by the novel system against the reference system under three ventilation schemes.

The tracer gas measurement data collected by the two systems under three different ventilation schemes were used to compute the respective air change rates using Equation (2). Figure 7 shows the air change rates detected by the novel system plotted against the reference system. The air change rates identified by the reference system and the novel system generally agreed with each other, with an average percentage error of 8% (1.9–14.4%).

### 3.3. Potential Application and Limitations

The novel R134a tracer-gas-sensing network demonstrated a fast response, high sensitivity, and consistency toward changes in R134a concentration. After calibration, the measurement values were consistent among receivers, and the system could detect sudden and explicit spatial and temporal variations in the tracer gas with high similarity. This sensing network can be an economical alternative to traditional tracer gas systems for evaluating ventilation performance.

Compared to the highly accurate traditional tracer gas systems at a market price of USD 75,000, the developed tracer-gas-sensing network has a much lower development cost of around USD 250 and several advantageous features, including wireless data transfer, high mobility and flexibility, quick response, unlimited sampling points, short sampling cycling, comparable accuracy, real-time remote access, control, and data visualization. The sensing network can identify the spatial distribution of the tracer gas to evaluate air mixing and find areas with poor ventilation. It can also be adopted to simulate the movement and dispersion of small-size particles driven by airflow. Since the system can be controlled remotely through the web-based tracer gas system control platform, this application is especially useful in studying the dispersion patterns of airborne bioaerosols and toxic gases and potentially determining the transmission risks in indoor environments unsuitable for human occupancy. 

One of the drawbacks of this novel system is the adaptation of immediate wireless data transmission without internal data storage. In that context, if the Wi-Fi is disconnected, the measurement data will be lost and can no longer be retrieved. This lack of “fail-safe” backup storage results in a need for data imputation. Fortunately, this inadequacy can be easily improved using a microcontroller with an external memory storage function that temporarily stores the measurement data and transfers them to the cloud once the Wi-Fi connection is restored. Moreover, the recent development of ESP-MESH network architecture enables multiple devices to form a communication network such that data packages can reach the cloud server without all being connected to the access point. This advancement can minimize data package loss and enable a sizeable physical coverage beyond the access point Wi-Fi coverage [46]. Aside from the data loss issue, since the sampling start time of the receiver depends on the time when the receivers of the sensing network are switched on and connected to the Wi-Fi, when there is a need for pairwise comparison between receivers, the discrete temporal data of the receivers cannot be synchronized without data manipulation and post-processing. An identical drawback can be found in the traditional tracer gas system as it takes turns to sample the six points. However, given the novel system’s short sampling cycle of 10 s, the effect of time lapse on measurement data should be minimal. 

As previously mentioned, the lack of an internal pump could lead to an unsteady flow rate through the tracer gas sensor of the novel receiver. However, the sensor has a high sensitivity to variations in levels of R134a. If the indoor environment is relatively stagnant, the diffusion of air into the device and thus through the sensor can be limited by low airflow. Similarly, a concentrated tracer gas can remain in the device without the “flushing” function that pushes out any residue tracer gas inside the device if the airflow is not high enough. However, this feature provides a more detailed variation of the tracer gas concentration influenced by ventilation, which is advantageous when the system is employed to study the dispersion patterns and transmission risk of airborne bioaerosols and toxic gases.

## 4. Conclusions

Existing IoT applications in indoor air mainly assess the levels of IAQ pollutants in indoor environments for general monitoring. On the other hand, this study proposed a novel application for evaluating airflow patterns and ventilation performance for experimental purposes. Built upon an existing IoT system, a novel IoT-enabled wireless tracer-gas-sensing network with a new sensor application was developed. Its performance was assessed by comparing it with traditional tracer gas measuring instruments. When deployed in an indoor environment served by mechanical ventilation, the experimental results showed that the novel sensing network could provide detailed spatial and temporal profiles of the tracer gas that were compatible and highly correlated to those produced by a traditional tracer gas system. Within the sensor detection range, a high correlation between the R134a levels measured by the nearby novel receivers was observed with R^2^ = 0.93–0.99, suggesting the sensing network’s consistent and conforming performance. Compared to the reference system, this system has a much quicker response in detecting the changes in tracer gas levels and a comparable accuracy. Regarding ventilation performance evaluation, the novel system successfully identified the air change rates with an average percentage error of 8%. As it has a fast response and is wireless and highly sensitive, with consistency among receivers, this novel tracer-gas-sensing network can not only be an affordable alternative to traditional tracer gas systems for evaluating ventilation performance but is also a valuable tool for building engineers, infection control experts, and epidemiologists in studying the dispersion of airborne bioaerosols and toxic gases, enacting infection risk control and management. Future research on developing low-cost, high-accuracy R134a sensors could further improve the performance of the sensing network.

## Figures and Tables

**Table 1:** The alignment costs of the warping paths of time-series data collected by the novel system, Receiver B and Receiver C, in the dynamic time-warping analysis.

Receiver C	Receiver B					
	S1	S2	S3	S4	S5	S6
S1	**22.2**	1074.5	1395.2	1526.8	1038.8	361.7
S2	2982.0	1123.6	1568.9	1961.7	2051.8	2746.9
S3	1329.6	537.0	**140.6**	**136.5**	**222.6**	926.1
S4	2610.2	738.7	338.3	**322.8**	796.9	2206.7
S5	632.4	606.8	533.8	667.3	**225.9**	**160.3**
S6	342.1	733.0	876.1	1128.0	543.7	**47.5**

Data with low alignment costs are highlighted in bold.

**Table 2:** Correlation coefficients between data collected by the reference system (A) and the novel system receivers (B and C). Boxes filled with grey colour are the correlation coefficients between data collected at the exact sampling location. The coefficients highlighted in bold suggest a high correlation.

	S1-A	S1-B	S1-C	S2-A	S2-B	S2-C	S3-A	S3-B	S3-C	S4-A	S4-B	S4-C	S5-A	S5-B	S5-C	S6-A	S6-B	S6-C
S1-A	**1.000**	**0.652**	**0.725**	−0.258	−0.258	−0.263	−0.196	−0.278	−0.250	0.155	0.003	0.048	0.282	0.166	0.202	0.698	−0.616	0.112
S1-B	**0.652**	**1.000**	**0.854**	−0.217	−0.217	−0.218	−0.172	−0.209	−0.209	0.120	0.051	0.073	0.187	0.129	0.160	0.446	−0.378	0.138
S1-C	**0.725**	**0.854**	**1.000**	−0.224	−0.228	−0.228	−0.206	−0.256	−0.229	0.066	−0.050	−0.016	0.228	0.150	0.195	0.444	−0.434	0.005
S2-A	−0.258	−0.217	−0.224	**1.000**	**0.998**	**1.000**	**0.889**	**0.918**	**0.979**	−0.318	−0.292	−0.320	−0.367	−0.331	−0.340	−0.155	0.508	−0.002
S2-B	−0.258	−0.217	−0.228	**0.998**	**1.000**	**0.999**	**0.912**	**0.938**	**0.989**	−0.268	−0.240	−0.269	−0.328	−0.286	−0.298	−0.175	0.490	−0.025
S2-C	−0.263	−0.218	−0.228	**1.000**	**0.999**	**1.000**	**0.893**	**0.922**	**0.981**	−0.311	−0.281	−0.311	−0.367	−0.328	−0.339	−0.159	0.512	0.000
S3-A	−0.196	−0.172	−0.206	**0.889**	**0.912**	**0.893**	**1.000**	**0.991**	**0.963**	0.147	0.163	0.139	−0.012	0.047	0.027	−0.242	0.297	−0.155
S3-B	−0.278	−0.209	−0.256	**0.918**	**0.938**	**0.922**	**0.991**	**1.000**	**0.978**	0.064	0.109	0.076	−0.120	−0.041	−0.068	−0.281	0.416	−0.103
S3-C	−0.250	−0.209	−0.229	**0.979**	**0.989**	**0.981**	**0.963**	**0.978**	**1.000**	−0.123	−0.095	−0.124	−0.250	−0.202	−0.215	−0.194	0.457	−0.038
S4-A	0.155	0.120	0.066	−0.318	−0.268	−0.311	0.147	0.064	−0.123	**1.000**	**0.963**	**0.982**	**0.858**	**0.896**	**0.875**	−0.215	−0.578	−0.458
S4-B	0.003	0.051	−0.050	−0.292	−0.240	−0.281	0.163	0.109	−0.095	**0.963**	**1.000**	**0.996**	**0.706**	**0.797**	**0.754**	−0.291	−0.378	−0.335
S4-C	0.048	0.073	−0.016	−0.320	−0.269	−0.311	0.139	0.076	−0.124	**0.982**	**0.996**	**1.000**	**0.761**	**0.834**	**0.798**	−0.277	−0.452	−0.386
S5-A	0.282	0.187	0.228	−0.367	−0.328	−0.367	−0.012	−0.120	−0.250	**0.858**	**0.706**	**0.761**	**1.000**	**0.967**	**0.984**	−0.215	−0.781	−0.649
S5-B	0.166	0.129	0.150	−0.331	−0.286	−0.328	0.047	−0.041	−0.202	**0.896**	**0.797**	**0.834**	**0.967**	**1.000**	**0.993**	−0.366	−0.626	−0.663
S5-C	0.202	0.160	0.195	−0.340	−0.298	−0.339	0.027	−0.068	−0.215	**0.875**	**0.754**	**0.798**	**0.984**	**0.993**	**1.000**	−0.304	−0.679	−0.660
S6-A	0.698	0.446	0.444	−0.155	−0.175	−0.159	−0.242	−0.281	−0.194	−0.215	−0.291	−0.277	−0.215	−0.366	−0.304	**1.000**	−0.197	**0.743**
S6-B	−0.616	−0.378	−0.434	0.508	0.490	0.512	0.297	0.416	0.457	−0.578	−0.378	−0.452	−0.781	−0.626	−0.679	−0.197	**1.000**	0.426
S6-C	0.112	0.138	0.005	−0.002	−0.025	0.000	−0.155	−0.103	−0.038	−0.458	−0.335	−0.386	−0.649	−0.663	−0.660	**0.743**	0.426	**1.000**

## Data Availability

Data are available upon request.
